# Using Modelling to Disentangle the Relative Contributions of Zoonotic and Anthroponotic Transmission: The Case of Lassa Fever

**DOI:** 10.1371/journal.pntd.0003398

**Published:** 2015-01-08

**Authors:** Giovanni Lo Iacono, Andrew A. Cunningham, Elisabeth Fichet-Calvet, Robert F. Garry, Donald S. Grant, Sheik Humarr Khan, Melissa Leach, Lina M. Moses, John S. Schieffelin, Jeffrey G. Shaffer, Colleen T. Webb, James L. N. Wood

**Affiliations:** 1 Department of Veterinary Medicine, Disease Dynamics Unit, University of Cambridge, Cambridge, United Kingdom; 2 Institute of Zoology, Zoological Society of London, London, United Kingdom; 3 Bernhard-Nocht Institute of Tropical Medicine, Hamburg, Germany; 4 Department of Microbiology and Immunology, Tulane University, New Orleans, Louisiana, United States of America; 5 Broad Institute, Cambridge, Massachusetts, United States of America; 6 Zalgen Labs, LLC, Germantown, Maryland, United States of America; 7 Lassa Fever Program, Kenema Government Hospital, Kenema, Sierra Leone; 8 Institute of Development Studies, University of Sussex. Brighton, United Kingdom; 9 Sections of Infectious Disease, Departments of Pediatrics and Internal Medicine, School of Medicine, Tulane University, New Orleans, Louisiana, United States of America; 10 Department of Biostatistics and Bioinformatics, Tulane School of Public Health and Tropical Medicine, New Orleans, Louisiana, United States of America; 11 Department of Biology, Colorado State University, Fort Collins, Colorado, United States of America; CDC, United States of America

## Abstract

**Background:**

Zoonotic infections, which transmit from animals to humans, form the majority of new human pathogens. Following zoonotic transmission, the pathogen may already have, or may acquire, the ability to transmit from human to human. With infections such as Lassa fever (LF), an often fatal, rodent-borne, hemorrhagic fever common in areas of West Africa, rodent-to-rodent, rodent-to-human, human-to-human and even human-to-rodent transmission patterns are possible. Indeed, large hospital-related outbreaks have been reported. Estimating the proportion of transmission due to human-to-human routes and related patterns (*e.g.* existence of super-spreaders), in these scenarios is challenging, but essential for planned interventions.

**Methodology/Principal Findings:**

Here, we make use of an innovative modeling approach to analyze data from published outbreaks and the number of LF hospitalized patients to Kenema Government Hospital in Sierra Leone to estimate the likely contribution of human-to-human transmission. The analyses show that almost 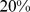 of the cases at KGH are secondary cases arising from human-to-human transmission. However, we found much of this transmission is associated with a disproportionally large impact of a few individuals (‘super-spreaders’), as we found only 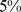 of human cases result in an effective reproduction number (*i.e.* the average number of secondary cases per infectious case) 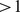, with a maximum value up to 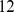.

**Conclusions/Significance:**

This work explains the discrepancy between the sizes of reported LF outbreaks and a clinical perception that human-to-human transmission is low. Future assessment of risks of LF and infection control guidelines should take into account the potentially large impact of super-spreaders in human-to-human transmission. Our work highlights several neglected topics in LF research, the occurrence and nature of super-spreading events and aspects of social behavior in transmission and detection.

## Author Summary

Many pathogens have the ability to infect different species. Lassa fever virus is an important example; this virus infects a species of rodent in West Africa, and can cause a severe disease in people. Lassa fever virus is transmitted from rodent-to-rodent, rodent-to-human, human-to-human and perhaps human-to-rodent. So far, the relative importance of these routes has not been assessed. Here we focus on the risk for humans; undoubtedly, most human infections are acquired by contact with rodents or their urine, but the relative risk of rodent-to-human and human-to-human transmission is unknown. We use mathematical modeling to address this. First, we identified Lassa fever outbreaks known to be due to human-to-human chains of transmission. Then, we looked at people hospitalized with the disease in Kenema Government Hospital, Sierra Leone (KGH), who could have been infected either by rodents or humans. We asked, what should the proportion of patients be who get infected by humans, assuming the statistical patterns observed in the human-to-human chains are the same in both instances? We found that around  of patients with Lassa fever in KGH probably acquired the disease from another person. In addition, the patterns of disease in people suggest that these  of cases are probably initiated by only a small number of infected people (who could be thought of as super-spreaders).

## Introduction

Diseases at the animal-human interface are in general subjected to different modes of cross-species transmission: animal-to-animal, animal-to-human, human-to-human and even human-to-animal. Estimating the relative contribution of each is of fundamental importance for the planning and implementation of appropriate infection control and preventive measures. This can be an extremely difficult task if humans and animals share the same physical space, and/or if experimentation (*e.g.* to quantify the probability of animal-to-animal transmission) is subjected to serious limitations. This is the case of Lassa fever (LF), a rodent-borne disease endemic in West Africa. Despite its clear zoonotic origin, there are strong arguments, listed below, to hypothesize that a significant proportion of the burden of LF in humans arises from human-to-human transmission. The aim of this work is to test whether or not patterns in the epidemic curve describing the cases of LF observed in Sierra Leone [1], are compatible with patterns observed in chains of pure human-to-human transmission recorded in nosocomial and extra-nosocomial outbreaks [2], [3].

Lassa fever is an acute, viral hemorrhagic disease caused by Lassa fever virus (LASV), an enveloped RNA virus of the Arenaviridae. The disease was first recognized in the village of Lassa, Nigeria in 1969, which caused the death of two missionary-nurses and the grave illness of a third [4]. However, cases consistent with LF from the eastern part of Sierra Leone can be traced back to 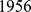 [5]. Since the identification of LASV, human-to-human transmission has been documented in several nosocomial outbreaks (*e.g.* [2], [3] and also [6] for a review), leading to an initial perception that the virus was both highly contagious and virulent [4]; this resulted in stringent requirements for containment of the patients [7]. Soon after, its zoonotic origin was recognized and *Mastomys natalensis*, one of the most common African rodents, was identified as the reservoir of the virus [8]. The risk of nosocomial transmission was shown to be dramatically reduced by using simple barrier nursing method [7], [9]–[11], suggesting that the risk of human-to-human transmission might be negligible.

These findings support an apparent, modern-day consensus that in the epidemiology of the disease, human-to-human transmission plays a less important role compared to zoonotic transmission. Accordingly, it has been suggested that patients with LF in non-endemic countries should not be confined to biosafety level 4 containment [10], and patient containment guidelines issued by the Centers for Disease Control (CDC) and the UK Department of Health and Social Security, in the past have been amended to be less restrictive (see [11], [12], also [13] and its previous versions).

**Figure 1:**
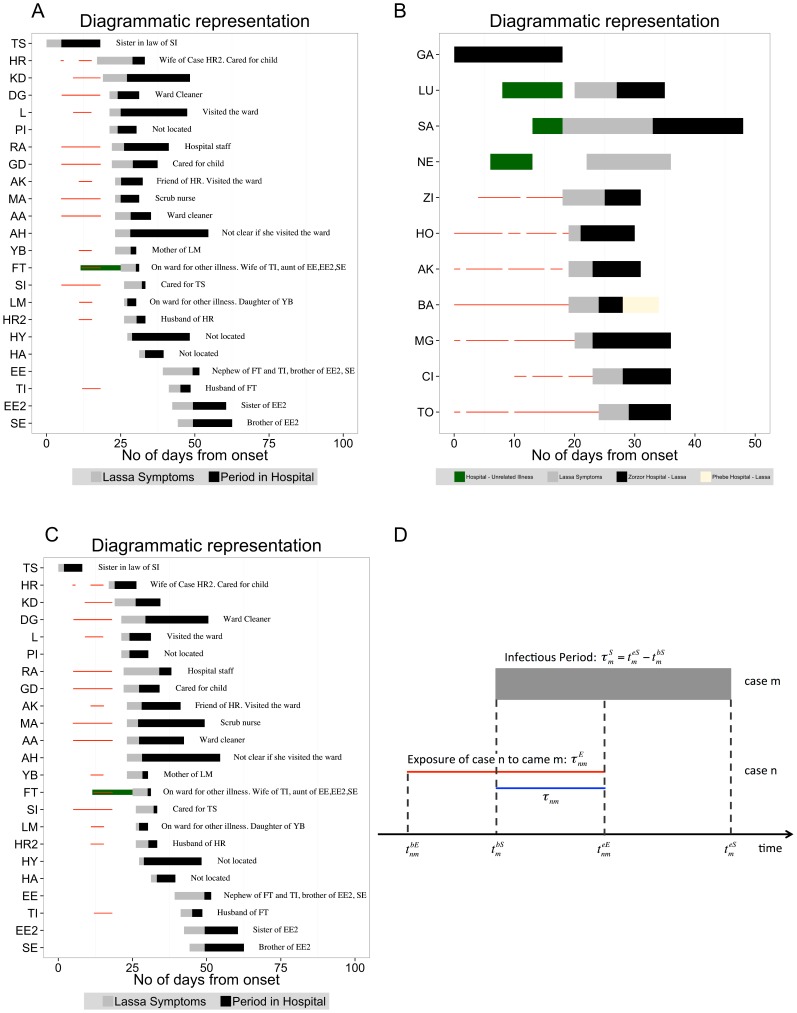
Nosocomial outbreaks. A: Diagrammatic representation of LF cases admitted at Jos Hospital, Nigeria (total duration of the outbreak 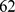 days), showing period of illness and interrelation among patients [2]. The horizontal bars represent each patient. The x-axis is the time expressed in days from the start of the outbreak, when TS developed the illness (thus time 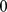 in the calculation corresponds to 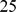 December 1969). The grey portion of the bars are the period between the onset of the symptoms and admission to hospital; the black portion of the bars are the period between admission to hospital and discharge/death of the patients; the red thin lines are the period of exposure to the index case TS. The green bar represent the time when the patient was at the ward for unrelated illness. Note, the same diagram in [2] present an extra case, JT, which is not included here. This case refers to Dr. Jeanette M. Troup one of the first scientists working on Lassa Fever Virus, who contracted the disease from an autopsy accident incurred during examination of one of the fatal cases. B: Diagrammatic representation of LF cases admitted at Zorzor Hospital (total duration of the outbreak 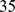 days), Liberia, showing period of illness and interrelation among patients [3]. C: As in Fig. 1.A, but the periods of illness (symptoms plus time at hospital) are randomly permuted. The contact network is kept the same. D: An example of how the time 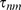 was calculated. In this particular case 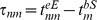 if 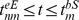 and 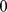 otherwise, where 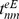 is the time when case 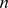 is no longer exposed to case 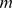.

This narrative concerning the relative importance of human-to-human transmission for LASV, however, requires re-evaluation as there are important indications of human-to-human transmission. More precisely, one of the early nosocomial outbreaks, in Jos, Nigeria (see [2], Fig. 1 and also the Supporting Information, S2 Text) was triggered by an index case that transmitted to possibly 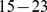 others in the hospital, with no indication of iatrogenic transfer. Further cases of extra-hospital transmission within a single family (five from the same family, 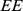, 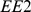, 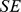, 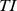, and 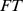 who likely initiated the chain) were reported, here and throughout we refer to this chain as an ‘extra-nosocomial’ chain. Haas *et al.* [14] investigated secondary transmission after an imported case of LF into Europe and found that one of 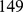 contacts that were tested serologically, a physician who examined the patient on day 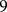 of illness, had become infected. The authors concluded that, during the initial phase of symptomatic LF the risk of transmission is low, but it may increase with progression of disease and increasing viral excretion. Emond *et al.* [15] described a case of LF in the UK in which the virus was isolated from urine 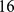 days after the acute phase had ended, despite not being detected earlier. The virus may also be found in pharyngeal secretions for 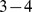 weeks after the onset of clinical signs [16]. In an experimental model, Stephenson *et al.* [17] showed the ability to infect guinea pigs and cynomolgus monkeys with LASV via the respiratory route and Peters *et al.* [18] demonstrated fatal LASV transmission to monkeys through being held in the same room for 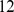 days with inoculated rodents. Sagripanti *et al.* [19], in a dark room at ambient laboratory temperatures controlled between 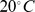 and 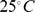 and 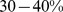 relative humidity, showed that the time required for a 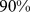 reduction in viral load of LASV in glass containers was 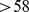 hours and was 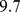 days for a 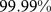 relative humidity. Also, Kernéis *et al.* [20] identified that risk factors for positive seroconversion to LASV included either having received a medical injection, or having lived with someone displaying a haemorrhage, in the previous twelve months. No factors related to contact with rodents were identified. Similarly, McCormick *et al.* [21] reported a lack of correlation between human LASV-specific IgG prevalence and either the level of domestic infestation by *Mastomys*, or the presence of LASV infection in *Mastomys*. These observations, taken together, suggest that a significant (if perhaps variable) proportion of the burden of LF might be associated with human-to-human transmission.

Estimating the contribution of human-to-human transmission of LASV and related patterns of transmission (*e.g.* existence of super-spreaders) is of fundamental importance when considering risk assessment and control of LF and related diseases such as the one caused by the arenavirus, Lujo virus [22], not least because LF is one of the more common haemorrhagic fevers exported from endemic areas [23]–[28]. In addition, perceiving LF as essentially a zoonotic disease only acquired from rodents with little or no infection arising from human beings, may have prevented investigations of the role, if any, of human-to-rodent transmission (i.e. spillback) in the epidemiology of LF.

Understanding routes of transmission and the proportion of LF cases resulting from human-to-human transmission is critical for developing and prioritizing effective prevention and control interventions, especially in the presence of large variation among subjects in their capability of infecting. The current Ebola outbreak has emphasised further the need for targeted biosecure measures which distinguish managing hemorrhagic fever cases and outbreaks from preventing spillover from reservoirs. This issue has not previously been fully addressed for Lassa Fever. Traditional approaches, such as cluster analysis, to distinguish human-to-human transmission from pure zoonotic transmission cannot be employed here due to the potential of clustering of infection in households from common exposure to infected rodents in addition to clustering arising from infected people. We overcome this problem by adapting other approaches [29], [30], and using data from nosocomial and extra-nosocomial outbreaks and hospitalized patients in Kenema Governmental Hospital (KGH), Sierra Leone [1]. We use these data to provide an estimation of the contribution of human-to-human transmission to the Lassa fever occurrence in endemic areas and to provide a more-robust assessment of the risk of secondary spread from index cases.

## Materials and Methods

### Ethics statement

The Tulane University Institutional Review Board and the Sierra Leone Ethics Committee approved the research [1]. Patients either approached KGH directly or were referred to the KGH Lassa Ward from regional health centers or the hospital's general ward on the basis of suspicion of LF. All adult subjects provided written informed consent for the analysis and publication of anonymized laboratory and clinical data. A parent or guardian of any child participant provided written informed consent on their behalf. All data were anonymized prior to analysis.

### Structure and rationale of the modeling approach

**Figure 2:**
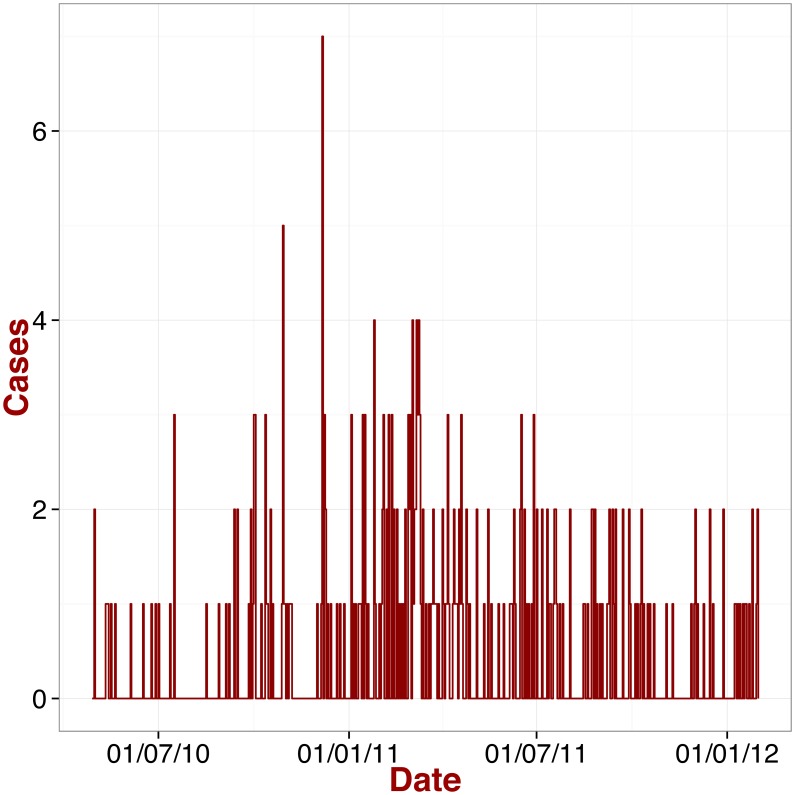
Epidemic curve. Daily number of referred/visiting patients at KGH (confirmed cases only) from the 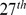 of April 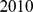 to the 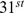 of January 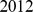, [1].

- Carey, Monath and co-workers [2], [3] provided evidence of nosocomial and extra-nosocomial chains that are examples of human-to-human transmission of LASV. Based on these early works[2], [3] and on the arguments listed in the introduction, it is reasonable to hypothesize that a proportion 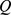 of hospitalized patients in KGH (Fig. 2) contracted the disease from a human source (see section “Available data and evidence of human-to-human transmission”).
- The next step was to estimate this proportion 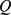 ensuring that aspects of (*e.g.* the effective reproduction number) the epidemic curve from KGH are compatible with those in the observed chains [2], [3].
- To this end, we employed and re-adapted the method of Wallinga and Teunis [29], who developed a method to calculate the effective reproduction number (which takes into account depletion of susceptibles) for an epidemic curve. If the network of transmission is known, no further information is required (see section “The effective reproduction number in the nosocomial and extra-nosocomial outbreaks”). Otherwise the distribution of the generation time, *i.e.* the time between a primary case and a secondary case, needs to be ascertained (see section “The effective reproduction number for cases of hospitalised patients in KGH”).
- An important feature of the approach of [29] is the option to consider a fraction of cases in the curve as externally imported, *i.e.* people being infected outside the community. In the context of hospitalized patients in KGH, the cases from animal-to-human transmission are interpreted as externally imported cases, whose proportion is 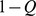. As we don't know which cases arises from zoonotic or human origin, we randomly selected a fraction 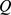 of the number of hospitalized patients at the Lassa ward and considered this subset of the epidemic curve as a pure human-to-human chain of transmission. This random sampling was repeated many times to ensure the findings are based on a reliable statistics.
- We calculated i) a daily mean effective reproduction numbers, 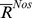 and 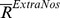 (definition below and in the glossary in S1 Text), associated with the nosocomial and extra-nosocomial chains of pure human-to-human transmission [2], [3] as well as the corresponding distributions of the generation times; ii) based on these distributions of the generation times we then estimated a daily mean effective reproduction numbers, 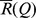, associated to each subset, consisting of a fraction 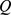 of patients, of the epidemic curve from KGH; iii) finally, by imposing equality of the two reproduction numbers: either 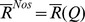 or 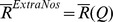, we estimated the proportion of cases arising from human-to-human transmission, 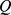.

#### Appropriateness of comparing data from Sierra Leone with the Jos/Zorzor outbreaks

Like Kenema in Sierra Leone, Jos and Zorzor are at the heart of the Nigerian and Liberian endemic areas of LF occurrence and, as in Sierra Leone, it is assumed that LASV has been circulating in these areas historically [5], [31], [32]. This is supported by molecular evidence that the Nigerian LASV strain is ancestral to the Sierra Leonean one [33]. In addition, although studies in human prevalence of antibodies to LASV exhibit variation amongst regions (*e.g.* low values in coastal areas), in general human seroprevalence appears to be similar in Nigeria and in the Mano River region (Guinea, Sierra Leone, Liberia) [20], [34]–[40].

Furthermore in recent decades, infection control has focused essentially in minimizing nosocomial transmission, albeit with partial success [41]–[43]. In particular, a study by Tobin *et al.* [42] revealed a general lack of knowledge of barrier nursing among health workers in rural areas. This problem is expected to be even more persistent among non-professionals, suggesting that control measures have not significantly changed since LF was discovered. Hence despite these data sets being collected at quite different points in time, health-care practices have not changed meaningfully over this time period.

Finally, the Jos/Zorzor outbreaks were exceptionally severe disease outbreaks, therefore by analyzing only hospitalized patients in KGH, we ensured that we are comparing equivalent situations.

These considerations justify the choice of comparing the data from KGH with the extra-nosocomial outbreak that occurred in Jos, while the appropriateness of comparing the KGH data with the nosocomial cases in the Jos outbreak is one of the hypothesis being tested in the current work.

#### Available data and evidence of human-to-human transmission

We analyzed the data from two nosocomial LF outbreaks: Jos, Nigeria in 1970 (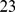 cases) [2] and Zorzor, Liberia in 1972 (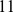 cases) [3]. In the Jos outbreak, extra hospital infections with no contact with the index case were observed (a single family 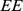, 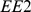, 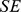, 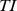, and 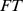 with the three children 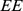, 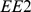 and 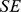 who never visited the ward). These appear to be human-to-human chains; sampling and testing of rodents near the homes of LF patients in Jos, as well as in the larger geographic area, showed no evidence of LASV in rodents [2], further supporting human-to-human transmission maintaining these epidemics. Further details of the two outbreaks and the full networks of contacts are presented in the Supporting Information, S2 Text.

In contrast with many emerging zoonoses, the reported incidence of LF in people is high in endemic areas, as reflected in data from KGH in Sierra Leone [1] (Fig. 2), thus allowing a more robust analysis of the transmission dynamics. KGH is the only health facility in Sierra Leone where people can be diagnosed and treated for suspected LF. The hospital facilities include an isolation ward specifically for LF patients with a highly trained clinical staff equipped with appropriate personal protective equipment. KGH records provide hospitalized patient data (day of admission, day of discharge, etc.) for suspected and confirmed cases of LF, divided by age, gender, ethnic group, location and other factors [1].

We used data abstracted from patient medical charts and LF diagnostic tests for 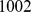 suspected Lassa cases presenting to the KGH Lassa Ward from 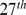 of April 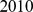 to the 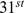 of January 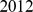. Among these subjects, 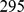 (Fig. 2) were confirmed as LF cases, *i.e.* either subjects with acute infection (tested positive LF using an antigen-based ELISA approach) or with recent LF (tested positive to IgM antibodies) [1]. These data correspond to the most accurate and complete set of patient records available at KGH. April 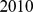 was chosen as the lower endpoint for our study sample due to significant improvements in data quality. These improvements are largely attributed to several NIH-funded research projects to develop and improve the diagnostic tests for LF. Improvements in clinical data quality can be attributed to more comprehensive questionnaire forms and increased community outreach and surveillance activities.

#### The effective reproduction number in the nosocomial and extra-nosocomial outbreaks

We calculated the effective reproduction number based on the observed dates of onset of symptoms, start and duration of exposure to the index cases and start and duration of exposure to all other cases. This information was directly obtained from the literature [2], [3], which provided detailed descriptions of the network of contacts (Fig. 1 and S1 table in S2 Text). Inspired by the work of Wallinga and Teunis [29], the relative likelihood 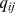 that case 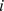 has been infected by case 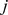 was calculated as:
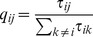
(1)here 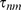 is the time of exposure of case 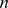 to case 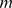 while case 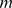 is infectious, *i.e.* the interval 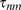 is given by the intersection 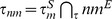, where 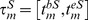 is the infectious period of case 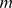, calculated as the difference between the time 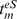, when the symptoms end (either because the patient recovers or dies) and the time 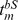, when the symptoms begin; 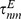 is the duration of exposure of case 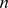 to case 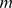 provided by the literature [2], [3]. It is important to note that 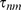, and thus 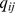, is explicitly time-dependent, (Fig. 1.D). The index 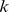 represents all possible cases within a nosocomial outbreak. Important underlying assumptions are: each event can independently start a new chain of human-to-human transmission, beginning of infectiousness coincides with the onset of symptoms, and infections occur with equal probability at any time during the interval 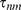. Accordingly, the sum 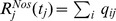 over all cases 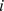, represents the *individual* effective reproduction number for case 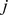 at the time 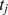 when the case arises. If multiple cases are observed at the same time 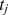, then 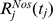 is averaged appropriately. To increase the sample size and improve the estimation, the duration of the symptoms of LF and the period spent at the hospital associated with each patient were randomly rearranged among the 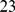 cases (Fig. 1.C), then the *ensemble* average individual effective reproduction number was calculated based on 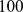 of these permutations. The network of contacts was kept the same. The identical approach was used to calculate the effective reproduction number, 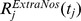, for the extra-nosocomial situation, with the network of contacts restricted to the family 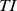, 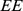, 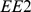, 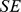 and 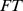 who presumably initiated the chain. We use the notation 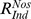 and 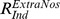 to indicate the set of all individual effective reproduction numbers for the nosocomial and extra-nosocomial cases respectively, while the total effective reproduction numbers 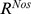 and 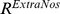 represent the corresponding average number of cases during the entire outbreak, *i.e.* 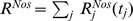 and 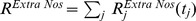 respectively. To adjust for the different duration expected in different outbreaks, we calculate a *daily mean* effective reproduction number as 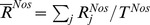 and 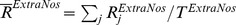 where 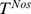 and 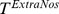 are the typical duration of the nosocomial and extra-nosocomial outbreaks.

The correctness of the approach was corroborated by performing the same analysis but by imposing that each case is exposed only to the index case. For the situation in Jos, the individual reproduction numbers were zero for all cases except for 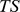 which results in 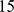, *i.e.* the only cases exposed to 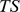 and marked with a thin red line in Fig. 1.

**Figure 3:**
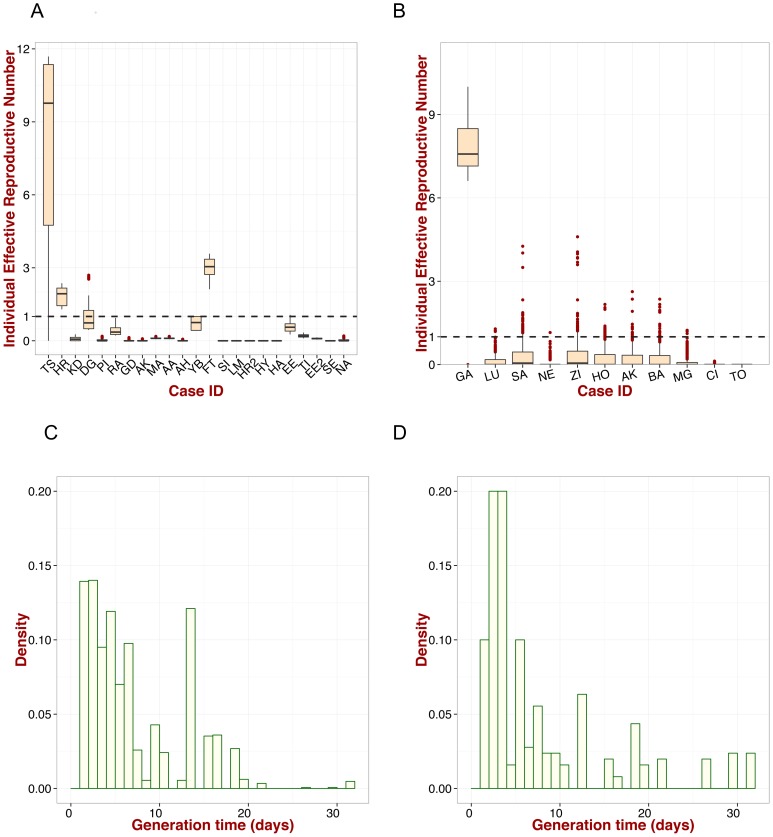
Individual effective reproduction number and generation time. Box-plot for the individual 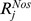 for the nosocomial outbreak described in [2] based on the 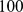 permutations of the duration of illness. It shows the first and third percentiles, the minimum and maximum values, the median, and outliers (red dots). The dashed line represents the case when the effective reproduction number is equal to 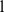. A: nosocomial outbreak in Jos [2]. B: nosocomial outbreak in Zorzor [3]. C: Distribution of generation time for the two nosocomial outbreaks. The statistics are based on the 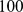 permutations of the duration of illness. D: Distribution of generation time for extra-nosocomial cases. The statistics are based on the 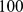 permutations of the duration of illness.

The distribution of the quantity 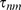 is interpreted as the distribution of the generation time, *i.e.* the time between a primary case and a secondary case, and it is presented in Figs. 3.C and 3.D (see also Figures S2, S3, S4 and S5 in S2 Text).

#### The effective reproduction number for cases of hospitalised patients in KGH

Following the approach of Wallinga and Teunis (see [29], [30] and their appendix for validation of the procedure), the relative likelihood that case 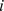 has been infected by case 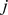, given their difference in time of symptom onset 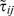, approximated here as the difference in time of admission to hospital, is then the likelihood that case 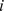 has been infected by case 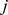, normalized by the likelihood that case 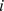 has been infected by any other case 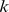


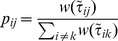
(2)where 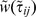 is the distribution for the generation interval and it is assumed to be the empirical distribution obtained from the nosocomial and extra-nosocomial outbreaks (shown in Figs. 3.C and 3.D). The effective reproduction number for case 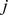 is the sum over all cases 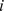, weighted by the relative likelihood that case 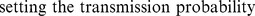 has been infected by case 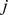


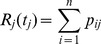
(3)


This quantity depends on the time 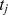 when case 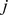 occurs. The set of all 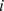 cases are obtained by the epidemic curve, describing the daily number of reported cases by date of symptom onset (Fig. 2), 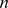 is the total number of reported cases. Underlying this calculation is the assumption that the spread of the disease occurs through human-to-human transmission only, however, a substantial proportion of cases, 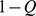, is expected to be due to zoonotic transmission, *i.e.* only through contacts with the rodent population, which can be considered as cases that have contracted infection from outside the population. These, therefore, cannot be regarded as secondary-tertiary etc. cases, although they can initiate a human-to-human chain. Under these circumstances, the effective reproduction number in equation (3) must be calculated by removing these 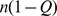 imported cases, *i.e.*

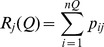
(4)


The set of all individual effective reproduction numbers is indicated by 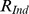, while 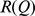 is the *total* effective reproduction number, *i.e.* 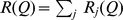, where to simplify the notation we dropped the dependency on 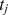. This value represents the average number of cases during the entire epidemic compatible with the particular generation time. As above, we averaged to obtain the *daily mean* effective reproduction number 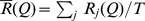 where 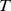 is the duration of the epidemics. The calculations were carried out by using the R package R0, provided by Obadia and co-authors [30], [44].

We compared two published outbreaks to illustrate the likely bounds of human to human transmission. Initially, we assumed that the extent of human-to-human transmission in nosocomial outbreaks in Jos and Zorzor [2], [3] represent the general situation in an endemic area. Alternatively, we rejected this assumption and considered only the out of hospital human-to-human transmission in Jos (five from the same family, 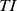, 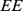, 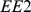, 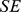 and  who initiated the chain), as being representative of the general endemic situation. We estimated the generation number and the mean nosocomial reproduction numbers for these two alternatives. By imposing equality with the mean nosocomial reproduction numbers (either 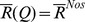 or 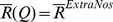), we inferred the proportion 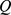.

To allow comparison with the effective reproduction number for the Kenema data, which by definition is based only on outbreaks where the primary case is assumed to be among those reported [29], the index patients 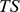 and 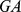 from the Jos and Zorzor outbreaks were assumed to be secondary cases to unreported human cases. On the grounds of realism, and also computational economy, the epidemic curve from KGH (Fig. 2) is assumed to be a collection of multiple chains of mean duration 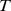, rather than a 2-year long un-interrupted epidemic. The starting times of each human-to-human chain were randomly chosen within the 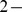 year period of the KGH epidemic curve. Similarly, as humans and rodents share the same physical space, cross-species transmission can occur at any time, thus the 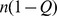 imported cases were randomly chosen from the network of contacts. For each value of 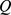, the ensemble sample of the simulations was 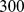.

## Results

### Effective reproduction number for nosocomial outbreaks

Figs. 3.A and 3.B show the effective reproduction number for each patient for the two nosocomial outbreaks respectively. As expected, the largest values are associated with the index case 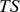 and 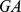 for the Jos and Zorzor outbreaks, respectively. In several cases, however, the effective reproduction number 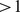; particularly important is case 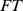 in the Jos outbreak with an effective reproduction number 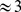, who most likely initiated extra-nosocomial transmission in her family.

### Estimating the contribution of human-to-human transmission

**Figure 4:**
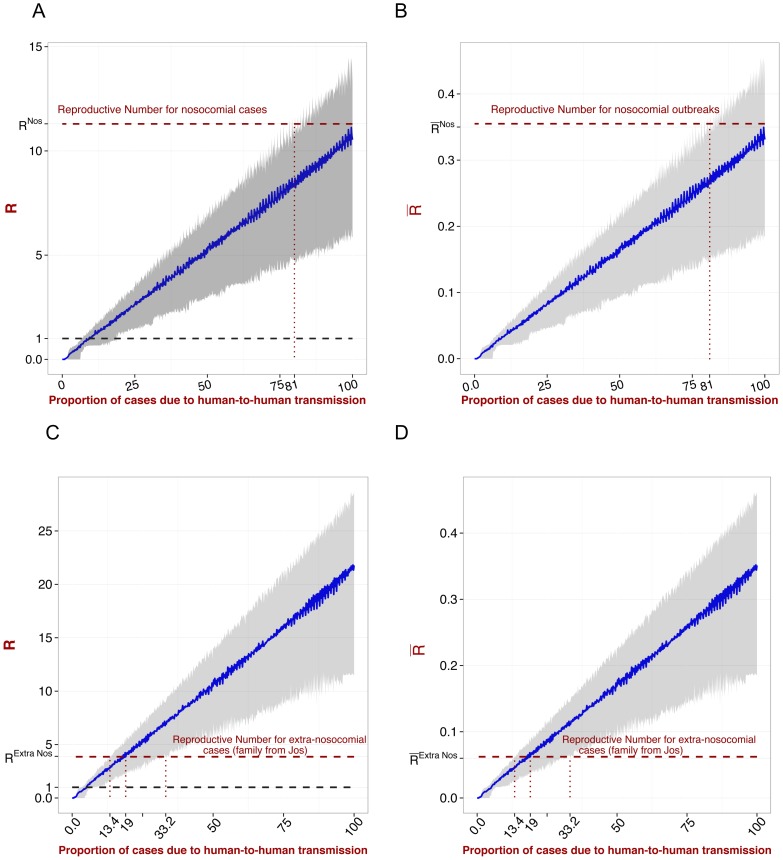
Contribution of human-to-human transmission. Mean value of the total effective reproduction number, 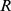 and its daily mean, 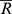, for the KGH epidemic curve *vs* the proportion 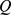 of cases due to human-to-human transmission (blue line). The shaded grey area covers the range between the 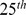 and 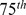 percentiles in 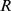 and/or 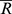; the dashed red line represents the mean, nosocomial, effective reproduction number. A and B: 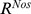 and 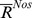 based on the full networks (in Jos and in Zorzor) of nosocomial cases; 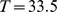 days. C and D: 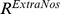 and 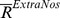 based on the extra-nosocomial cases in Jos; 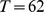 days.

Fig. 4 shows the total effective reproduction number 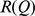 and its daily mean 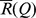 for the cases in KGH, *vs* the estimated proportion 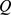 of cases due to human-to-human transmission. The shaded grey area covers the range between 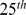 and 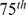 percentiles arising from the 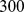 simulations for each value of 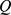. The predictions were then compared with the total effective reproduction number (or with the equivalent daily mean), in the nosocomial outbreaks 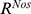 (or 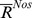) based on the full network of cases and with the extra-nosocomial cases in Jos alone 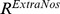 (or 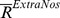). For the full network of cases, the mean nosocomial reproduction number was higher than the mean KGH one, implying that the severe hospital outbreaks ought to be seen as exceptional circumstances. In contrast, the daily mean effective reproduction number arising from the Jos extra-hospital cases (due only to human-to-human transmission) was entirely compatible with the daily mean KGH effective reproduction number if we allow a proportion of cases to be due to human-to-human transmission 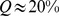. Based on the 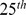 and 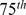 percentiles in the predictions for the reproduction number 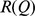, the lower and upper estimates for the proportion of human-to-human transmission are 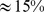 and 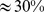 respectively.

### Quantifying the impact of the super-spreaders

**Figure 5:**
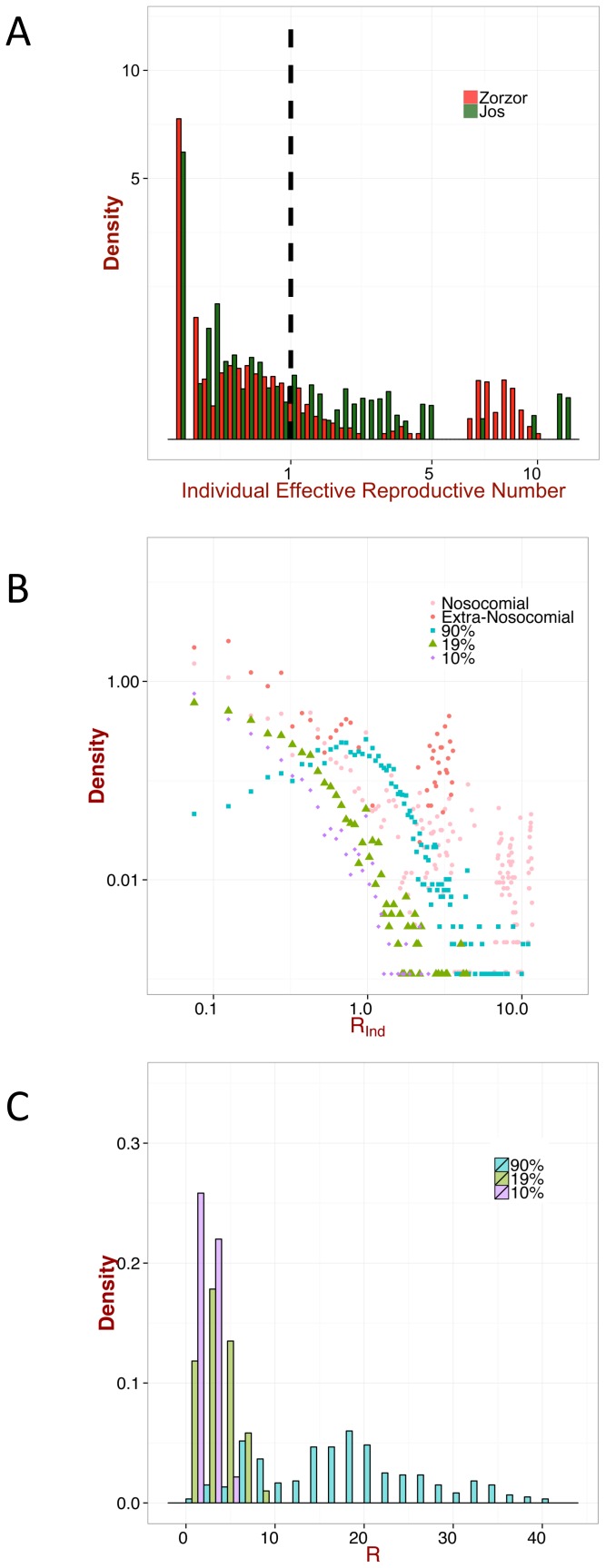
Impact of super-spreaders I. A: Distribution of all individual 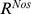 for both nosocomial outbreaks, based on the 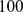 permutations of the duration of illness. Mean value of the joint data: 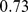, median: 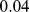, maximum: 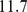, proportion of cases when 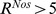: 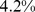, proportion of cases when 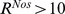: 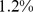. B: Distribution of the effective reproduction number for cases of hospitalized patients in KGH for different values of the contribution of human-to-human transmission, 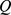, the corresponding data for the extra-nosocomial (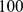 permutation based on 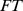, 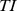, 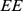, 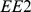, 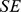 cases in Jos) and all nosocomial outbreaks (based on all Jos and Zorzor cases) are also shown. C: Distribution of the total effective reproduction number, *i.e.* the average number of cases during the entire duration of the epidemic for different values the contribution of human-to-human transmission, 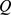.

Super-spreaders are individuals who can infect a disproportionately large pool of susceptibles [45]. Here, super-spreading events are identified and quantified by analyzing how the effective reproduction number is distributed. The distribution of the individual effective reproduction numbers for the Jos and Zorzor outbreaks, based on the 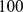 permutations of the duration of illness, is shown in Fig. 5.A. Although 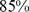 of the predictions for individual 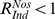, there is a fat tailed distribution, with extreme values of 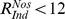. Similar patterns are observed for KGH cases for the individual effective reproduction number 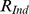. As shown in Fig. 5.B and 5.C, the distribution of the individual and total, effective reproduction numbers, 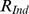 and 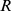 appears to have a fat-tailed distribution, especially for larger values of 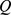.

**Figure 6:**
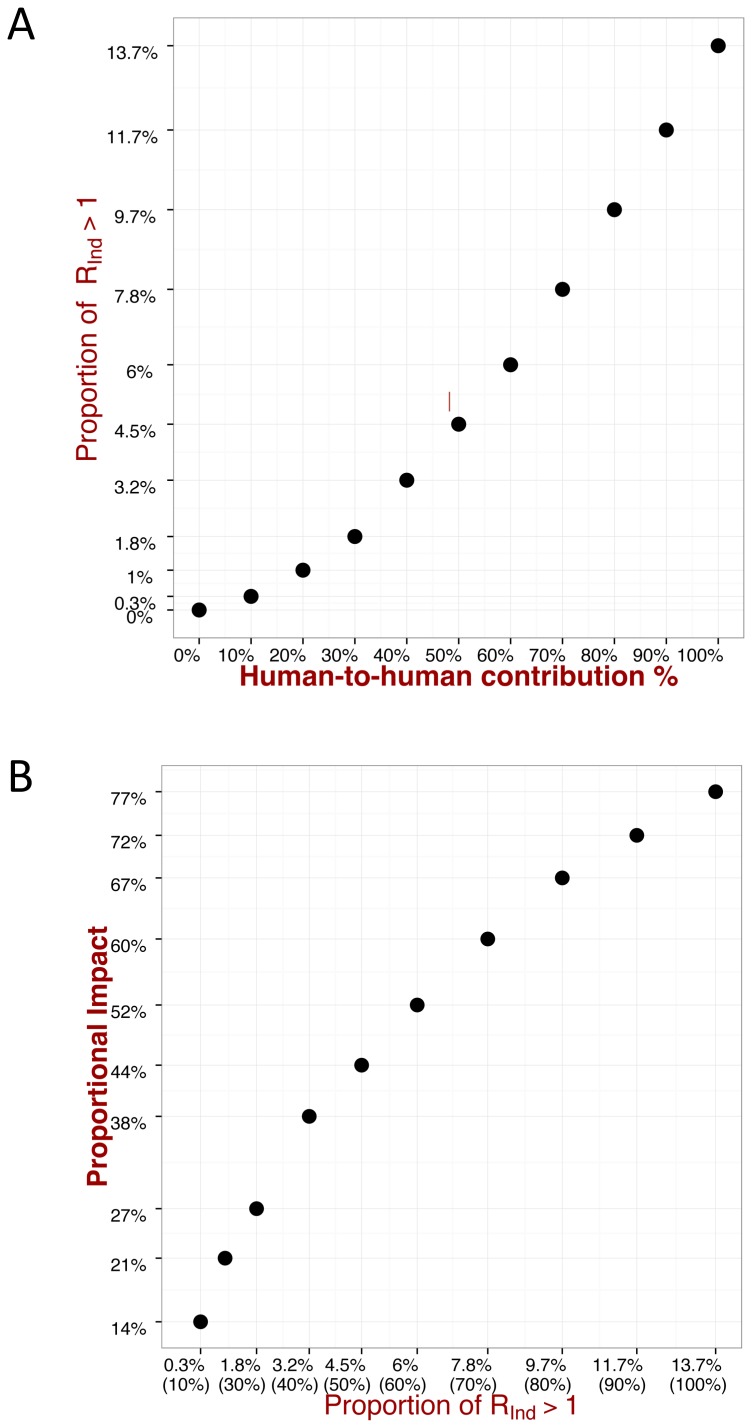
Impact of super-spreaders II. A: proportion of cases when the individual effective reproduction number 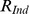 is greater than one. (*i.e.* the ratio of the cardinalities of 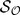 and 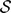, where 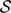 is set of all simulated 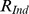 and 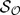 the subset of cases when 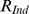 is greater than one). B: the expected, relative number of cases generated by this proportion. (*i.e.* the fraction of the areas of 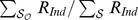)

A simple approach to evaluate the risk of super-spreaders is to invoke the so-called ‘20/80 rule’ (whereby 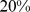 of cases cause 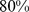 of transmission, see [45], [46]). To this end, for different values of the contribution of human-to-human transmission, 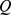, we calculated i) the proportion of cases when 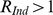 (Fig. 6.A), and ii) its proportional impact, given by the expected, relative number of secondary cases generated by this proportion (see Fig. 6.B for further explanations); the maximum 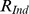 in the simulations was also recorded. For a contribution of human-to-human transmission in the region of 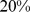, only 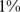 of realizations gave 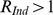, but they are, on average, responsible for 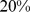 of secondary cases, with a maximum 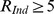. In an extreme situation, when the disease is transmitted only by humans, 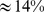 of cases are responsible for the 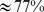 of secondary cases with a maximum 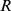 up to 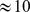, which resembles the ‘20/80 rule’.

More sophisticated ways to assess the risk of super-spreaders are presented in the S2 Text. In particular, we fitted the distribution for the individual effective reproduction number 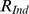 from KGH with an exponential and a log-normal distribution. The observed KGH distribution is ‘fatter’ than the corresponding exponential fit, although not as heavy as the log-normal. Furthermore we fitted two standard discrete distributions, the Poisson, representative of thin-tailed distributions, and the negative-binomial, representative of fat-tailed distributions, with the distribution for the integer part (as we are considering discrete distributions) of 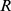, *i.e.* the average number of cases during the entire epidemic. As can be seen, the last distribution is better fitted by a negative-binomial distribution, especially for the tail (S6 Figure in S2 Text).

### Sensitivity of the predictions

#### Effect of the duration of epidemics

Different values for the typical duration of a human-to-human chain, 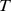, were explored. As expected, the longer the duration, 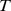, the larger the availability of susceptibles and the higher the effective reproduction number. However, when the effective reproduction number is rescaled by the duration of the epidemics, *i.e.* the daily mean effective reproduction number 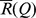, all the predictions are similar. This can be seen by comparing the plots 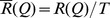 in Figs. 4.B (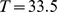 days), 4.D (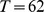 days) and S1 Figure in S2 Text (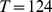 days).

Based on this invariance, we can infer the proportion 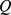, irrespective of the choice of the mean duration 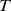, provided that we know the typical duration of the nosocomial and extra-nosocomial chains 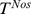 and 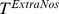. For the nosocomial case, a reasonable choice is 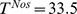 days, which is the average duration of nosocomial outbreaks observed in Jos and Zorzor hospitals (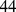 and 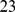 days respectively, the last days correspond to when the last person developed symptoms). For the extra-nosocomial chains the choice is less clear, as the member of the family were already in contact with the index case 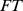 before she developed symptoms, and shedding of the virus could happen any time before symptom onset. Therefore we made a conservative choice of 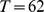 days which corresponds to the maximum duration of the Jos outbreak (here the last day corresponds to when case 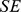 recovered). Any shorter choice of the duration 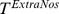, will result in a larger value of the proportion 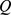 (for instance if the typical duration of the outbreak is 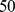 days, *i.e.* including while 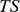 was at the hospital, we found that 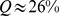 instead of 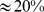 while the estimates based on the intersection with the 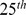-percentile and 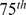-percentile were 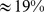 and 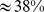 respectively rather than 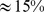 and 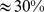), which reinforces the key message of this current work that a significant proportion of cases of LF arises from human-to-human transmission.

#### Effect of the distribution of the generation times

The robustness of the approach was tested by considering different distributions for the generation time. We considered i) the distribution of generation time arising from the entire network, ii) the distribution arising from the particular subset of the data corresponding to the extra-nosocomial cases 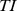, 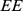, 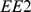, 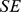 and 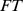, iii) a gamma distribution with the same empirical mean and variance, iv) the empirical distribution by removing part of the tail, v) a ‘stretched’ distribution by multiplying the empirical generation times by a factor 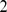 to allow for a longer shedding of the virus, and vi) a ‘shrunk’ distribution by multiplying the empirical generation times by a factor 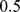. In general, each of these methods had limited effects on the predictions (see S2 Figure in S2 Text, S3 Figure in S2 Text, S4 Figure in S2 Text, and S5 Figure in S2 Text).

## Discussion

Disentangling the contribution of different hosts in spreading a zoonotic, emerging disease is a key challenge for determining effective, proportionate public health and safety measures. Such a conundrum has steered a scientific debate on LF, which appears to fluctuate around whether or not human-to-human transmission plays a major role compared to rodent-to-human transmission. The current work reconciles these two opposing paradigms. Here, we adopted a relatively simple mathematical approach to analyze data of hospitalized patients in KGH, Sierra Leone. The daily mean effective reproduction numbers, 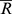, observed in the nosocomial outbreaks (only human-to-human transmission) are much larger, and thus incompatible, with the ones estimated from the data from KGH, even if we assume 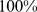 human-to-human transmission. If we regard the extra-nosocomial cases observed in the Jos outbreak as representative of disease transmission in an endemic area, then a significant proportion of LF cases (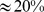) arise from human-to-human transmission. A significant proportion of these secondary cases, however, are attributable to a few events with disproportionately large effective reproduction numbers: super-spreading events. In general, the distributions of the individual reproductive number, 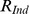, and the average number of cases during an epidemic, 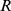, exhibits a tail heavier than the exponential or Poisson distributions, here used as benchmarks for thin-tailed distributions. This reveal over-dispersion indicating the presence of super-spreading events.

These results have implications for clinical practice and policy. Super-spreading occurrences appear not to be exceptional for infectious diseases [46], [47]. According to this perspective, the lack of recorded secondary cases in Britain after the importation of confirmed cases of LF [7], [12] should not be regarded as proof of absence of human-to-human transmission. Any future assessment of the associated risk of LF and formulation of patient containment guidelines [13], therefore, should take into account the fat-tail nature of the underlying distribution of individual reproduction number.

Super-spreading events have been documented for many infectious diseases [45], [46], including tuberculosis [48], measles [49] and SARS [50]–[52]. The underlying reasons for super-spreading are not fully understood, but include amount of pathogen excreted, length of the infectious period, social behavior and environmental factors. Understanding the mechanisms of super-spreaders in LF requires an understanding of all of these factors, but transmission of LASV is not well characterized. In the Jos outbreak, Carey et al. [2] speculated that the severe pulmonary involvement of the index case and the location of her bed could cause airborne spread of virus to the rest of the ward. The long persistence of viruria, even during the recovery period, could facilitate transmission of LASV to other people or to the rodent reservoir host, especially in rural settlements in areas of West Africa where sanitary facilities are limited. Participatory modeling and ethnographic research [53] would be an invaluable tool to assess the variety of practices and settings in which people come into contact with each other's urine and other body fluids, perceptions of risk, and approaches to hygiene. Apart the work of Stephenson *et al.* [17] and Peter *et al.* [18], we are not aware of specific experiments aimed to test routes of LASV transmission. Studies on environmental contamination are also required to explore if human-to-rodent transmission is important for the maintenance or spread of LASV infection [6].

### Limitations of this study and future work

The current model is based on the assumption that the distribution of the generation times observed in the extra-nosocomial outbreak is representative of the generation times in the Sierra Leonean situations. This is probably reasonable, but public health measures may impact on this in the future. The predictions are based on the assumption of uniform mixing, *i.e.* each case from KGH is potentially in contact with each other case. Although this is a reasonable assumption, considering the large human mobility in Sierra Leone, for livelihoods, work and trade, social visits and events [54], it will overestimate 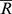. In addition, there is likely to be incomplete reporting which will underestimate 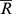. Although the two factors might compensate for each other, if KGH could reduce reporting bias and increase information on network contacts, it would be highly beneficial to studies such as this, especially as human mobility and overcrowding has been associated with an amplification of LF [31]. This represents a further area where participatory modeling/ethnographic research is much needed to gather information on actual patterns of mobility and social networking, and hence potential contact patterns.

Other methods are possible, for example cluster-based inference of the reproduction number (see *e.g.* [55], [56]) is a promising approach. In the current context, Kernéis *et al.* [20] provided detailed information on prevalence and risk factors of Lassa (*e.g.* history of collecting, cutting and eating rats) stratified by age. In addition, the age distribution from KGH is also available. These data could be combined together to build a matrix of transmission rates among age-groups (as done in [55], [56]) and between rodents and each age-group with values based on the findings of Kernéis *et al.* [20].

For the KGH data, the difference in time of onset of symptoms was approximated here with the difference in time of visit/referral to the hospital following disease onset. This assumption can be an important source of error as patterns of health seeking behavior might vary largely among the Sierra Leonean population. For example, a particular group of the population might favor traditional medicine and approach institutional health care only at a late stage of the disease. Patients from rural areas might be subjected to further delay due to poor infrastructure. Health seeking behavior is perhaps one the most fruitful areas where participatory modeling and ethnographic research have been successfully employed and should be considered in the present context.

The estimations here are based on the assumption that probability of cross-species transmission occurs at random throughout outbreaks, although these events are expected to be strongly driven by a multitude of interacting causes, including ecological (*e.g.* seasonality in the abundance of the reservoir), epidemiological (*e.g.* seasonality in the prevalence of the pathogen), genetic variation (*e.g.* broad set of pathogen life histories) and socio-economic (*e.g.* the practice of burning the fields after harvesting affecting the ecology and dispersal patterns of *M. natalensis*) factors.

The estimation of the proportion of human-to-human transmission was based on the assumption that the typical duration of a chain of transmission is equal to the duration of the extra-nosocomial outbreak. As this information was only approximately known, we made the prudent choice that the duration of the extra-nosocomial outbreak was the entire extent of the outbreak in Jos, which was the maximum value from the data available. This led to a conservative estimation of 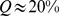. Despite some inevitable inaccuracies, all of our alternative/additional tests confirm that a significant proportion (in the region of 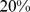 or greater) of the burden of LF is due to human-to-human transmission.

The basis of our work is that, to the best of our knowledge, the nosocomial and extra-nosocomial outbreaks in Jos and Zorzor were instances of pure human-to-human chains. This is a message too important to be neglected. Despite some improvement in the implementation of barrier nursing in hospital structures, there is no evidence that in the last decades prophylactic measures have significantly changed in ordinary situations as in households and villages, and this issue has been abundantly clear during the current Ebola outbreak in West Africa. It is therefore reasonable that chains of human-to-human transmission, like the extra-nosocomial outbreak that occurred in Jos more than 40 years ago are much more common than expected.

This study highlights the need for integrated One Health approaches to model zoonoses more effectively in order to better-inform disease control and prevention. Zoonoses remain a neglected group of diseases, under-prioritised in national and international health systems.

## Supporting Information

S1 Text List of symbols and glossary.
(PDF)

S2 Text Details on the nosocomial outbreaks and further tests.
(PDF)

S1 DataThe file provides the daily number of referred/visiting patients at KGH (confirmed cases only) from the 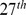 of April 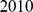 to the 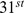 of January 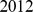 [1], as displayed in Fig. 2.(TXT)
