# Supplementary material for: Using Modelling to Disentangle the Relative Contributions of Zoonotic and Anthroponotic Transmission: The Case of Lassa Fever
Source: PLoS Negl Trop Dis. 2015 Jan 8;9(1):e3398. doi: 10.1371/journal.pntd.0003398 (PMC4288732; doi:10.1371/journal.pntd.0003398)
Supplement: S1 Text — List of symbols and glossary. (PDF) [file pntd.0003398.s001.pdf]

S1 Text. List of Symbols and Glossary

Extra nosocomial chain The cases of extra-hospital transmission within a single family (EE, $EE2$, SE, TI, and FT who likely initiated the chain) recorded in Jos, Nigeria in 1970 [2], Figure 1.A.

Generation time The time between a primary case and a secondary case.

Individual Effective Reproduction Number The average number of secondary cases arising from each single infectious case in a population made up of both susceptible and non-susceptible hosts.

KGH Acronym for Kenema Governmental Hospital, in Sierra Leone.

LASV Lassa fever virus.

LF Lassa fever.

Nosocomial chain The chain of nosocomial infections recorded in Jos, Nigeria in 1970 [2], Figure 1.A and in Zorzor, Liberia in 1972 [3], Figure 1.B.

Proportional impact The fraction of the areas of $\sum_{\mathcal{S}_{\mathcal{O}}} R_{Ind} / \sum_{\mathcal{S}} R_{Ind}$ where $\mathcal{S}$ is set of all simulated R_{Ind} and $\mathcal{S}_{\mathcal{O}}$ the subset of cases when $R_{Ind} > 1$.

Q Proportion of hospitalised patients in KGH (Figure 2), who contracted the disease from a human source. Estimating this value is a key goal of the current work.

$\mathbf{R_j^{Extra\ Nos}(t_j)}$ Individual effective reproduction number for case j at the time t_j for the extra-nosocomial situation, *i.e.* with the network of contacts restricted to the family TI, EE, $EE2$, SE and FT.

$\mathbf{R_{Ind}^{Extra\ Nos}}$. The set of all individual effective reproduction numbers for the extra-nosocomial situation.

$\mathbf{R^{Extra\ Nos}}$ Average number of cases during the entire outbreak for the extra-nosocomial situation, *i.e.* $R^{Extra\ Nos} = \sum_j R_j^{Extra\ Nos}(t_j)$.

$\mathbf{\overline{R}^{Extra\ Nos}}$ Daily mean effective reproduction numbers for the extra-nosocomial situations, *i.e.* $\overline{R}^{Extra\ Nos} = \sum_j R_j^{Extra\ Nos} / T^{Extra\ Nos}$.

$\mathbf{R_j^{Nos}(t_j)}$ Individual effective reproduction number for case j at the time t_j for the nosocomial situations, *i.e.* considering the entire network of contacts in Jos and Zorzor.

$\mathbf{R_{Ind}^{Nos}}$ The set of all individual effective reproduction numbers for the nosocomial situations.

$\mathbf{R^{Nos}}$ Average number of cases during the entire outbreak for the nosocomial situations, *i.e.* $R^{Nos} = \sum_j R_j^{Nos}(t_j)$.

$\mathbf{\overline{R}^{Nos}}$ Daily mean effective reproduction numbers for the nosocomial situations, *i.e.* $\overline{R}^{Nos} = \sum_j R_j^{Nos}/T^{Nos}$.

$\mathbf{R_j(Q)}$ The effective reproduction number for case j at the time t_j associated with a subset of the epidemic curve from KGH and assumed to be a pure human-to-human chain of transmission; this subset is obtained by randomly selecting a portion, of duration T, of the epidemic curve and then a fraction Q of the patients.

$\mathbf{R(Q)}$ The total effective reproduction number $R(Q) = \sum_j R_j(Q)$. This value represents the average number of cases during the entire epidemic compatible with the particular generation time.

$\mathbf{\overline{R}(Q)}$ The *daily mean* effective reproduction number $\overline{R}(Q) = \sum_j R_j(Q)/T$ where T is the duration of the epidemics.

T The epidemic curve from KGH (Figure 2) is assumed to be a collection of multiple chains of mean duration T, rather than a 2-year long un-interrupted epidemic.

$\mathbf{T^{Nos}}$ Typical duration of the nosocomial outbreaks.

$\mathbf{T^{Extra\ Nos}}$ Typical duration of the extra-nosocomial outbreaks.

$\tau_{\mathbf{nm}}$ The time of exposure of case n to case m while case m is infectious for the nosocomial and extra-nosocomial situations (Figure 1.D).

$\mathbf{q_{ij}}$ The relative likelihood that case i has been infected by case j for the nosocomial and extra-nosocomial situations.

$\mathbf{\tilde{w}}(\tilde{\tau}_{\mathbf{ij}})$ The distribution for the generation interval and it is assumed to be the empirical distribution obtained from the nosocomial and extra-nosocomial outbreaks (shown in Figures 3.C and 3.D).

$\mathbf{p_{ij}}$ The relative likelihood that case i has been infected by case j, given their difference in time of symptom onset $\tilde{\tau}_{ij}$, approximated here as the difference in time of admission to hospital.
