# Supplementary material for: Using Modelling to Disentangle the Relative Contributions of Zoonotic and Anthroponotic Transmission: The Case of Lassa Fever
Source: PLoS Negl Trop Dis. 2015 Jan 8;9(1):e3398. doi: 10.1371/journal.pntd.0003398 (PMC4288732; doi:10.1371/journal.pntd.0003398)
Supplement: S2 Text — Details on the nosocomial outbreaks and further tests. (PDF) [file pntd.0003398.s002.pdf]

S2 Text.

Nosocomial outbreaks in in Jos, Nigeria and in Zorzor, Liberia

Here we revisit the data of two hospital outbreaks of LF that occurred in Jos, Nigeria in 1970 [1] and in Zorzor, Liberia in 1972 [2] by looking at the effective reproduction number to estimate the severity of the diseases. A description of the Jos outbreak is presented below, while the full networks of contacts of 23 and 11 patients for the two respective outbreaks are presented in Figures 1.A and 1.B in the main text. For the Jos oubreak, TS is the index case, however other contacts are possible, for example case RA is a member of hospital staff. It is likely that this person was a source of exposure to other people working in the hospital, (*e.g.* the nurse MA and the cleaner AA) or spending a long time in the same ward (*e.g.* case FT, who was admitted to the ward for chronic renal disease). Extra hospital infections were plausible through case FT.

Description of nosocomial outbreaks in Jos

On 30 December 1969, person TS was admitted to ward A of Evangel Hospital in Jos, Nigeria due to a severe febrile illness (subsequently diagnosed as LF). TS was on the ward approximately 2 weeks. Both her new infant and a three year old daughter stayed with her during this period. Shortly after TS's discharge and return to Bassa, her mother and the two children became ill. The daughter died at home. A brother-in-law of TS, visited her while she was in the hospital and became ill too. Within two months another 23 people became ill, 16 of them were directly exposed to TS, while four other cases (EE, TI, EE2, SE) are probably instances of extra-hospital transmission within a single family as the three children EE, EE2, SE never visited the ward (see Figure 1.A in the main text). The most striking feature of the Jos outbreak is the apparent transmission of Lassa infection from one person to possibly 16 others exposed to her in the hospital ward. There is no indication of exchange of infected body-fluids such as blood-infected needles. According to [1]:

> *In one respect, the illness of TS did differ from most: there was severe pulmonary involvement. The original diagnosis had, in fact, been pneumonia. TS was, moreover, placed in a corner bed on ward A across which a prevailing breeze blew to the rest of the ward. Such a combination of factors could favour airborne spread of virus, but there is no firm basis for any conclusion.*

To support this hypothesis, Lassa virus was also isolated from the throat of patients who at the time had no objective signs of pharyngitis [3, 4].

Network of contacts

The possible network of contacts is presented in table S1. Co-presence at the ward included visitors caring for a relative and patients admitted for other illness. The period of exposure to the index case TS, is provided by Carey *et al.* [1] and it is represented by the thin red lines in Figure 1 (main text), for both the original and the randomly permuted networks. If the source of exposure is a friend/relative then the period of exposure is calculated since the source developed illness. If the source of exposure is a member of the hospital staff, then the period of exposure was calculated since the source developed illness with the condition that to the exposed case was at the ward during this time. If the source of exposure is a patient not related with the exposed case, then the period of exposure was calculated since the source was admitted to hospital with the condition that the exposed case was at the ward during this time. The time between when a patient developed illness and was admitted to the hospital can be inferred from Figure 1 (main text) for both the original and the randomly permuted networks. The three cases shaded in red (EE,EE2, and SE) were never at the hospital.

For the Zorzor outbreak the interrelations among patients were much simpler and can be inferred directly from the diagram in the original publication [2] and Figure 1.B (main text).

Table S1. Interrelation among patients in Jos outbreak. The initials in the first and third columns relate to the identities of individual patients.

**Exposed Case**	**Hospital Staff**	**Potential Sources Exposure**	**Relationship to Exposed Case**	**Potential Period of Exposure** (conditioned to the exposed case being at the ward during this time)
HR	No	TS	Ward Co-Presence	From Carey *et al.* [1]
KD	No	TS	Ward Co-Presence	From Carey *et al.* [1]
DG	Yes	TS	Ward Co-Presence	From Carey *et al.* [1]
L	No	TS	Ward Co-Presence	From Carey *et al.* [1]
RA	Yes	TS	Ward Co-Presence	From Carey *et al.* [1]
		DG	Work Colleague	Since DG developed illness
GD	No	TS	Ward Co-Presence	From Carey *et al.* [1]

Table S1. Interrelation among patients in Jos outbreak. (Con't)

**Exposed Case**	**Hospital Staff**	**Potential Sources Exposure**	**Relationship to Exposed Case**	**Potential Period of Exposure** (conditioned to the exposed case being at the ward during this time)
AK	No	TS	Ward Co-Presence	From Carey *et al.* [1]
		HR	Friend	Since HR developed illness
MA	Yes	TS	Ward Co-Presence	From Carey *et al.* [1]
		DG	Work Colleague	Since DG developed illness
		RA	Work Colleague	Since RA developed illness
AA	Yes	TS	Ward Co-Presence	From Carey *et al.* [1]
		DG	Work Colleague	Since DG developed illness
		RA	Work Colleague	Since RA developed illness
YB	No	TS	Ward Co-Presence	From Carey *et al.* [1]
FT	No	TS	Ward Co-Presence	From Carey *et al.* [1]
		HR	Ward Co-Presence	Since HR was admitted to hospital
		DG	Ward Co-Presence	Since DG developed illness
		L	Ward Co-Presence	Since L was admitted to hospital
		PI	Ward Co-Presence	Since PI was admitted to hospital
		RA	Ward Co-Presence	Since RA developed illness
		GD	Ward Co-Presence	Since GD was admitted to hospital
		AK	Ward Co-Presence	Since AK was admitted to hospital
		MA	Ward Co-Presence	Since MA developed illness
		AA	Ward Co-Presence	Since AA developed illness
		AH	Ward Co-Presence	Since AH was admitted to hospital

Table S1. Interrelation among patients in Jos outbreak. (Con't)

**Exposed Case**	**Hospital Staff**	**Potential Sources of Exposure**	**Relationship with Exposed Case**	**Potential Period of Exposure** (conditioned to the exposed case being at the ward during this time)
SI	No	TS	Brother-in-law	From Carey *et al.* [1]
LM	No	TS	Ward Co-Presence	From Carey *et al.* [1]
		YB	Daughter	Since YB developed illness
HR2	No	TS	Ward Co-Presence	From Carey *et al.* [1]
		HR	Husband	Entire time of HR illness
EE	No	FT	Nephew	Since FT developed illness
TI	No	TS	Ward Co-Presence	From Carey *et al.* [1]
		FT	Wife	Since FT developed illness
		EE	Nephew	Since EE developed illness
EE2	No	FT	Aunt	Since FT developed illness
		TI	Uncle	Since TI developed illness
		EE	Brother	Since EE developed illness
SE	No	FT	Aunt	Since FT developed illness
		TI	Uncle	Since TI developed illness
		EE	Brother	Since EE developed illness
		EE2	Sister	Since EE2 developed illness

Additional tests on the effects of the duration of epidemics and the distribution of the generation times

Effects of the duration of epidemics

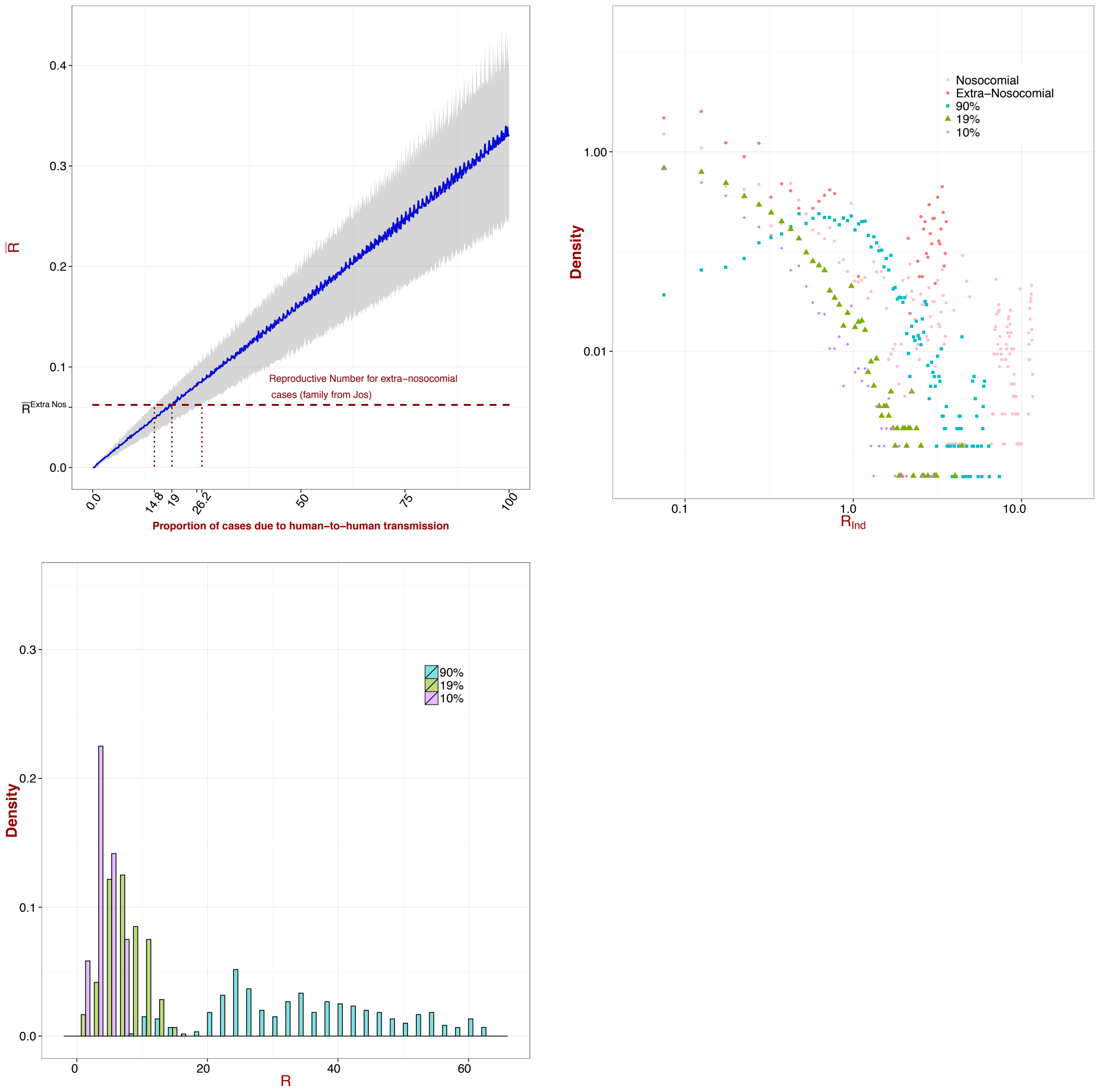

Figure S1. As in Figures 4.D, 5.B and 5.C, however the duration of each epidemic is assumed to be two times the mean duration of the Jos nosocomial outbreak (124 days instead of 62 days).

Effects of the distribution of the generation times

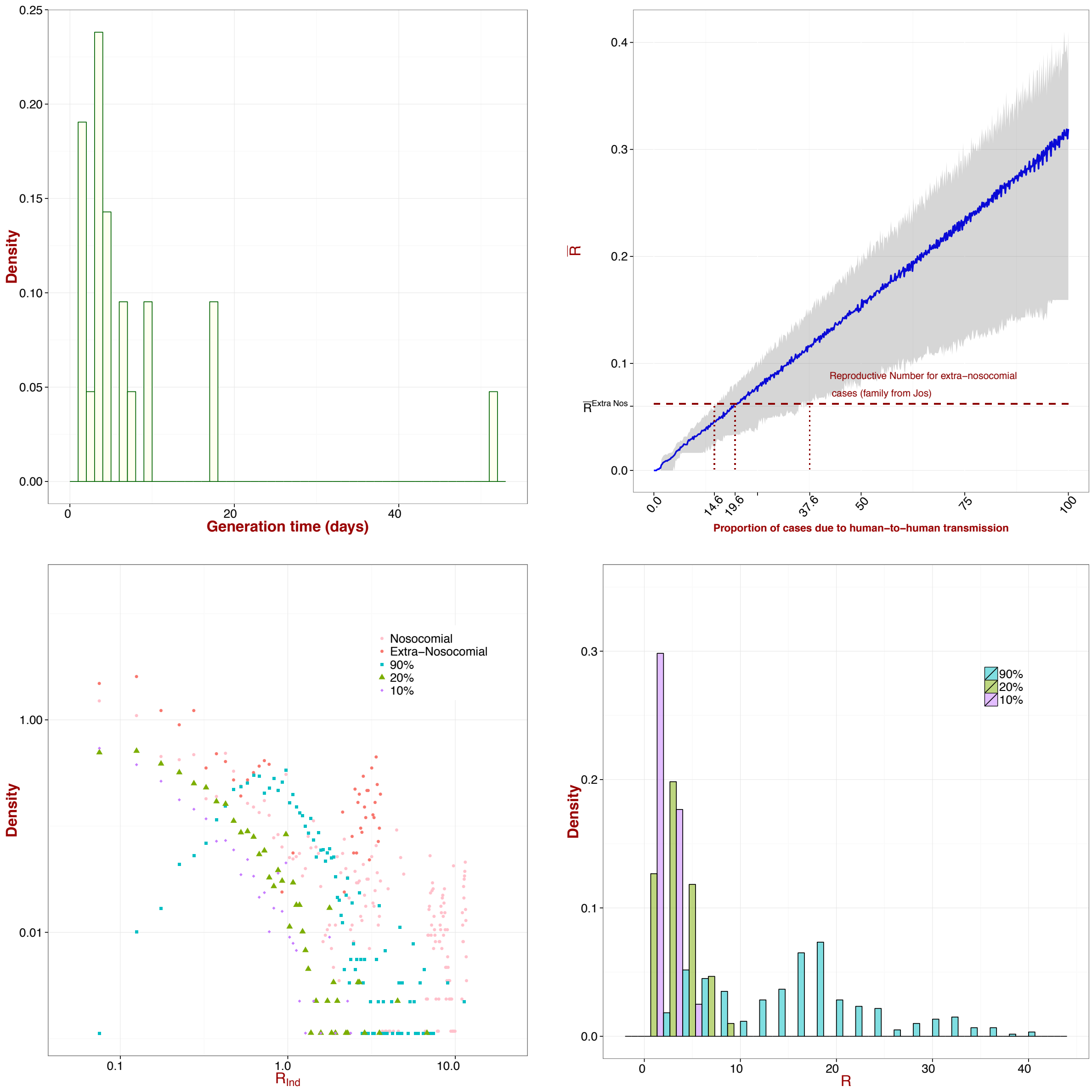

Figure S2. As in Figures 4.D, 5.B and 5.C, however the distribution of generation time is obtained by generating a gamma distribution with the same mean and variance as in the empirical distribution.

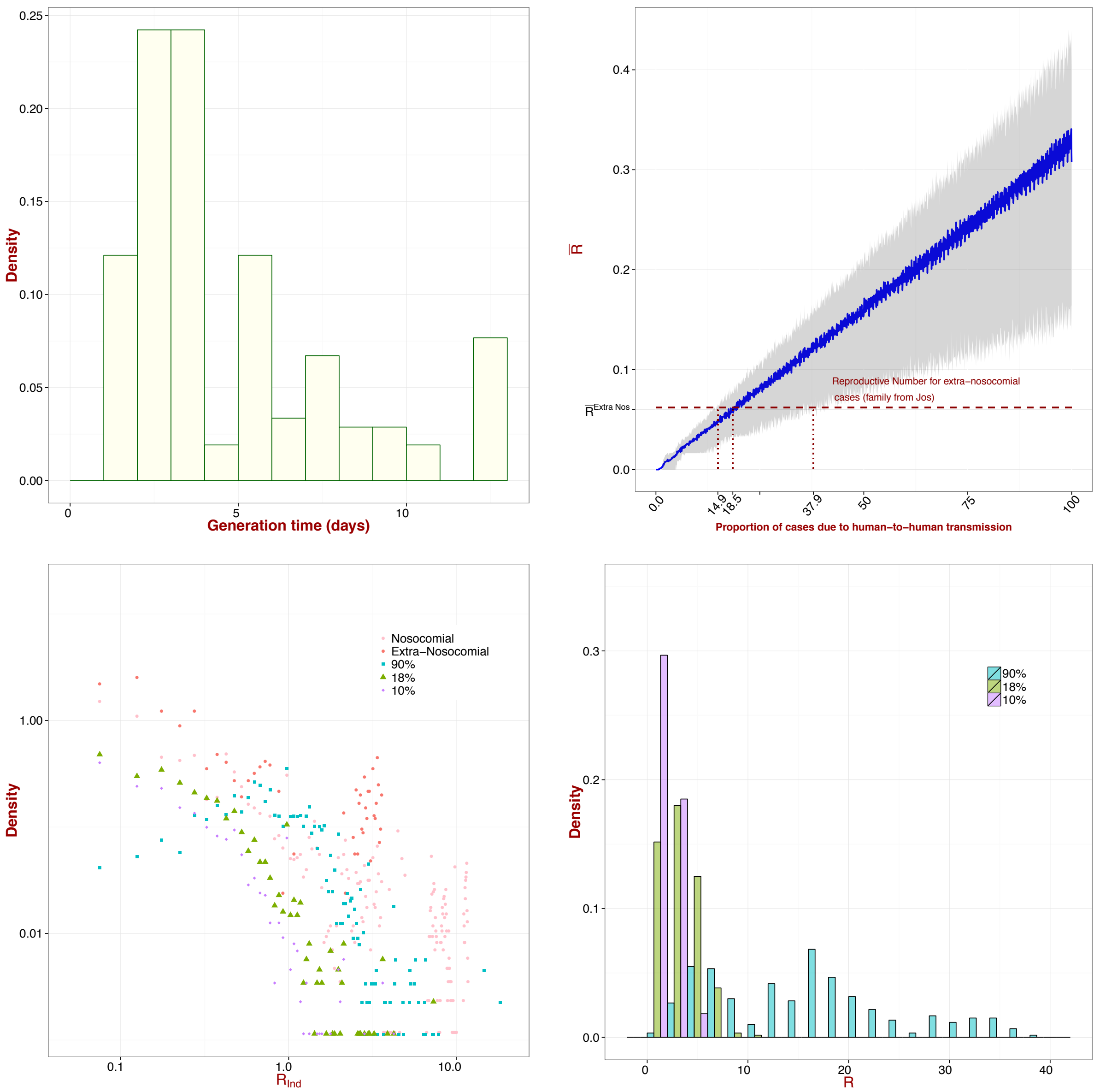

Figure S3. As in Figures 4.D, 5.B and 5.C, however the distribution of generation time (S3.A) is obtained by clipping the empirical distribution removing all generation times larger than 15 days.

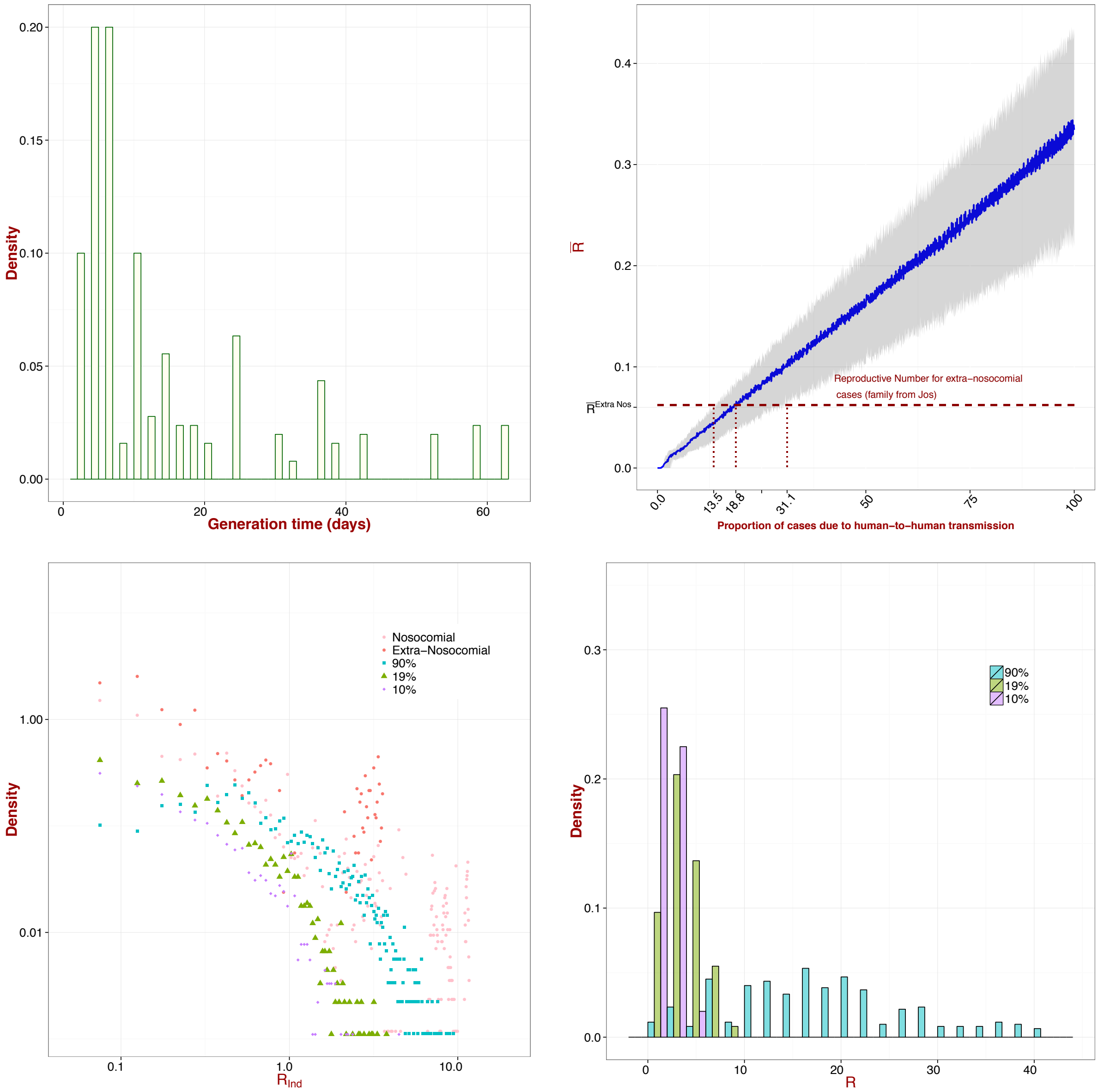

Figure S4. As in Figures 4.D, 5.B and 5.C, however the distribution of generation time (S4.A) is obtained by multiplying the empirical generation times by a factor 2 to mimic longer shedding of the virus.

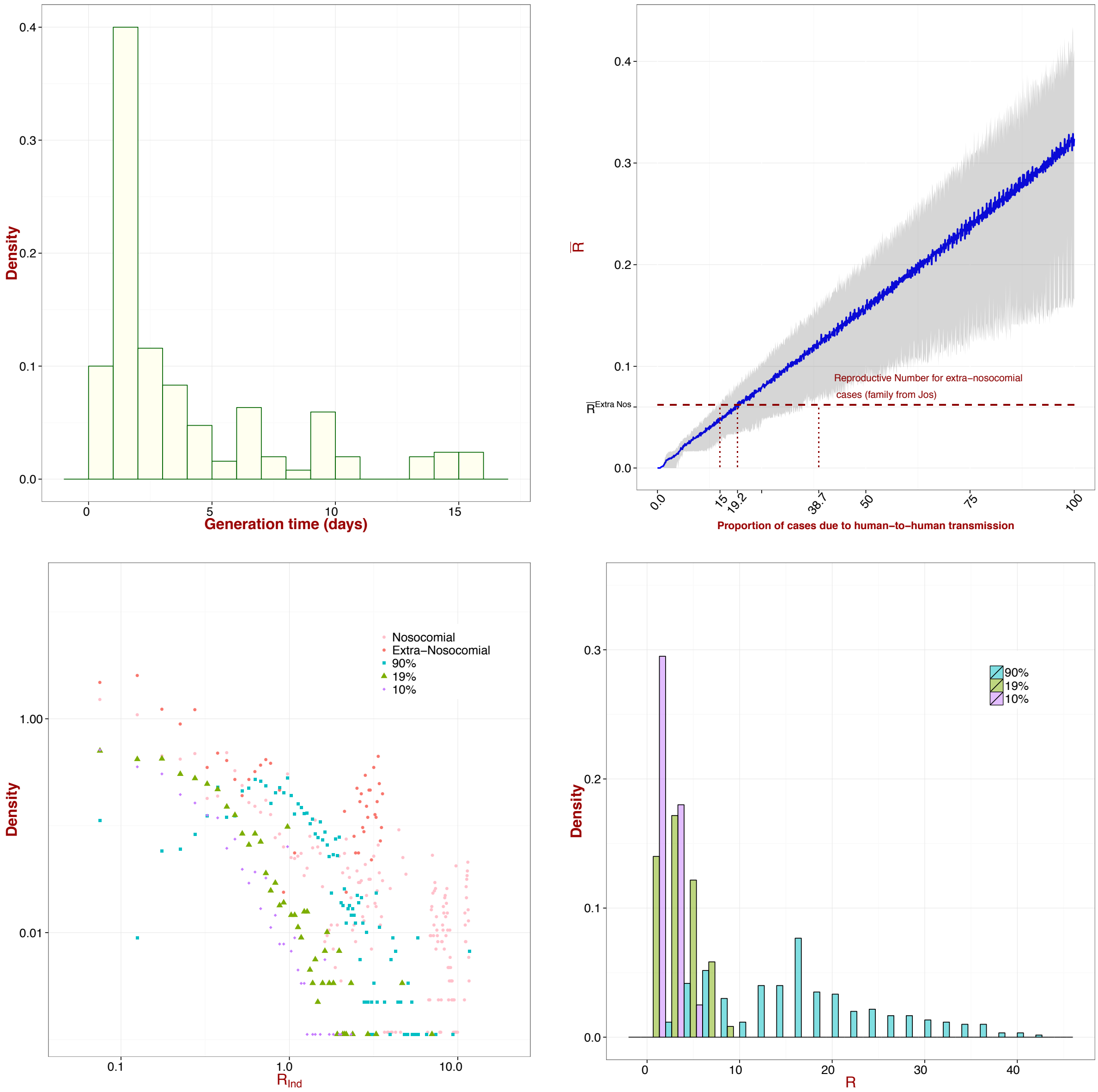

Figure S5. As in Figures 4.D, 5.B and 5.C, however the distribution of generation time (S5.A) is obtained by multiplying the empirical generation times by a factor 0.5 to mimic shorter shedding of the virus.

Further tests to quantify the impact of the super-spreaders

The impact of the super-spreaders is further clarified in Figure S6.A, which compares the observed distribution for the individual effective reproduction number, R_{Ind}, based on exponential and log-normal distributions respectively. The observed distribution lays between these two standard cases. For small contributions of human-to-human transmission (low Q), discerning whether or not the distribution of the individual effective reproduction number is described by a thin or fat tail function is difficult (Figure S6.B), this is not surprising as in the limiting case of $Q = 0$, the distribution should collapse to zero. However, for large values of the contribution to human-to-human transmission, a deviation from the Poisson case is evident (Figure S6.C) indicating that the average number of cases (the total effective reproduction number R) is intrinsically governed by fat-tailed distribution. The implication is that, although the mean effective reproduction number can be $<< 1$, the risk of super-spreaders is not negligible. These patterns suggest an occasional, but devastating, super-spreading event, when a minority of individuals can infect a large pool of susceptibles, can occur.

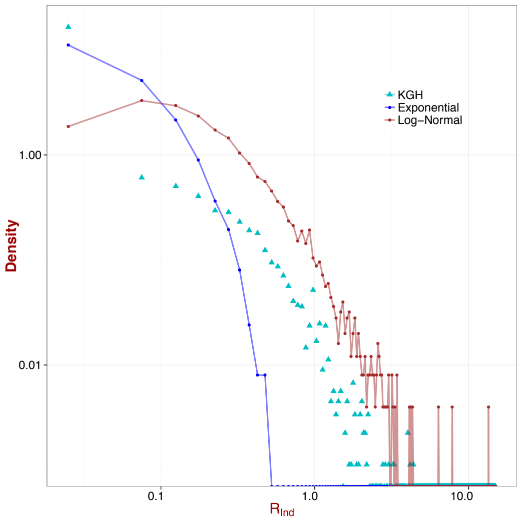

S6.A

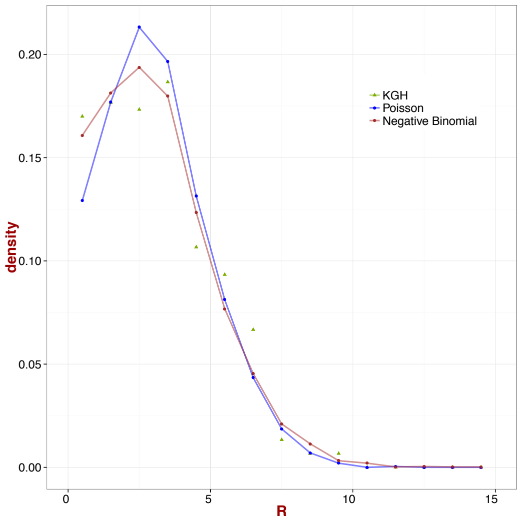

S6.B

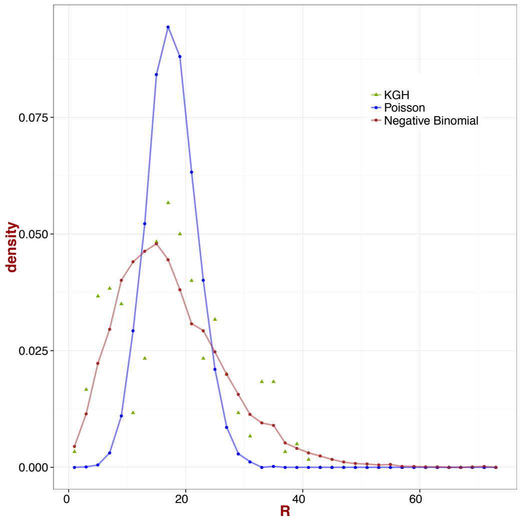

S6.C

Figure S6. S6.A Comparison of the simulated distribution for the effective reproduction number R with two exemplary distributions: the exponential and log-normal distributions, the contribution of human-to-human transmission, $Q = 19\%$. S6.B and S6.C: Comparison of the simulated distribution of the total effective reproduction number R, *i.e.* the average number of cases during the entire duration of the epidemic with two exemplary distributions for discrete values: a Poisson (thin-tail) and negative binomial distribution (fat-tail). S6.B: contribution of human-to-human transmission, $Q = 19\%$. S6.C: contribution of human-to-human transmission, $Q = 90\%$.

References

1. Carey D, Kemp G, White H, Pinneo L, Addy R, et al. (1972) Lassa fever Epidemiological aspects of the 1970 epidemic, Jos, Nigeria. Transactions of the Royal Society of Tropical Medicine and Hygiene 66: 402–408.

2. Monath TP, Mertens PE, Patton R, Moser CR, Baum JJ, et al. (1973) A hospital epidemic of Lassa fever in Zorzor, Liberia, March-April 1972. The American Journal of Tropical Medicine and Hygiene 22: 773–9.

3. Monath TP, Maher M, Casals J, Kissling RE, Cacciapuoti A (1974) Lassa fever in the eastern province of Sierra Leone, 1970-1972. II. Clinical observations and virological studies on selected hospital cases. The American Journal of Tropical Medicine and Hygiene 23: 1140–9.

4. Monath TP (1975) Lassa fever: review of epidemiology and epizootiology. Bulletin of the World Health Organization 52: 577–592.
